# Stability Determination of Intact Humanin-G with Characterizations of Oxidation and Dimerization Patterns

**DOI:** 10.3390/biom13030515

**Published:** 2023-03-11

**Authors:** Mustafa Ozgul, Anthony B. Nesburn, Nader Nasralla, Benjamin Katz, Enes Taylan, Baruch D. Kuppermann, Maria Cristina Kenney

**Affiliations:** 1 Department of Ophthalmology, Gavin Herbert Eye Institute, University of California Irvine, Irvine, CA 92617, USA; 2 Cedars-Sinai Medical Center, Los Angeles, CA 90048, USA; 3 IVD Technologies Inc., Santa Ana, CA 92705, USA; 4 Department of Chemistry, University of California Irvine, Irvine, CA 92697, USA; 5 Department of Obstetrics and Gynecology, University of California Los Angeles, Los Angeles, CA 90095, USA; 6 Department of Pathology and Laboratory Medicine, University of California Irvine, Irvine, CA 92617, USA

**Keywords:** peptides, stability, degradation products, oxidations, high-performance liquid chromatography (hplc), high-resolution mass spectrometry (hrms)

## Abstract

Humanin is the first identified mitochondrial-derived peptide. Humanin-G (HNG) is a variant of Humanin that has significantly higher cytoprotective properties. Here, we describe the stability features of HNG in different conditions and characterize HNG degradation, oxidation, and dimerization patterns over short-term and long-term periods. HNG solutions were prepared in high-performance liquid chromatography (HPLC) water or MO formulation and stored at either 4 °C or 37 °C. Stored HNG samples were analyzed using HPLC and high-resolution mass spectrometry (HRMS). Using HPLC, full-length HNG peptides in HPLC water decreased significantly with time and higher temperature, while HNG in MO formulation remained stable up to 95% at 4 °C on day 28. HNG peptides in HPLC water, phosphate-buffered saline (PBS) and MO formulation were incubated at 37 °C and analyzed at day 1, day 7 and day 14 using HRMS. Concentrations of full-length HNG peptide in HPLC water and PBS declined over time with a corresponding appearance of new peaks that increased over time. These new peaks were identified to be singly oxidized HNG, doubly oxidized HNG, homodimerized HNG, singly oxidized homodimerized HNG, and doubly oxidized homodimerized HNG. Our results may help researchers improve the experimental design to further understand the critical role of HNG in human diseases.

## 1. Introduction

Mitochondrial DNA (mtDNA) is double-stranded, circular DNA comprised of 16,569 nucleotide pairs that represents 37 genes encoding for 13 peptides, 22 transfer RNAs, and 2 ribosomal RNAs [1,2]. Mitochondria-derived peptides (MDPs), encoded by the human mtDNA, play essential roles in many cellular physiological processes that can affect aging and disease progression [3,4,5,6,7,8,9]. Exploring mitochondrial biology, several MDPs, consisting of 16–38 amino acids, have been identified [10]. Humanin (HN), the first identified MDP, contains 24 amino acids (2687.3 Da) [5] and has neuroprotective and anti-apoptotic properties in in vitro and in vivo models [5,11,12,13,14]. The serum HN levels decrease significantly with age and are associated with age-related diseases in rodent animal models and human clinical studies [15,16]. HN peptides protect against neurotoxicity in Alzheimer’s disease and suppress amyloid-beta-induced neuronal death in vitro [17]. The administration of exogenous HN peptides provides cytoprotective effects in Type-2 diabetes rat models [15] as well as myocardial and cerebral ischemia [18,19] and atherosclerosis [20] mouse models [3].

Humanin-G (HNG) is an HN derivative with a S14G substitution exhibiting 1000-fold more potent cytoprotective properties than HN, and it also demonstrates therapeutic potential for multiple diseases [5,21]. Similar to Humanin, HNG (2657.3 Da) inhibits amyloid-beta (Aβ)-induced death in primary neurons in vitro and demonstrates cytoprotective effects for myocardial ischemia-reperfusion injury in animal models [18,22,23,24]. HNG also has antitumor effects as shown in neuroblastoma tumor xenograft experiments [25]. The effect was linked to reduced angiogenesis and increased tumor cell apoptosis [25].

Recently, we investigated the effect of HNG in a transmitochondrial cybrid model for age-related macular degeneration (AMD), which is the most common cause of visual impairment in the elderly population. Cybrids are cell lines with identical nuclei but with mitochondria from different individuals with AMD or age-matched normal subjects. The AMD cybrids treated with HNG showed significantly increased levels of humanin receptor proteins and decreased levels of RNA/proteins involved in apoptosis, autophagy, and ER stress pathways [1]. HNG-treated AMD cybrids showed significantly lower levels of cell death and improved functions in vitro [1]. However, conducting in vivo studies has been challenging due to the instability of the HNG peptide because of its tendency to rapidly degrade, oxidize and dimerize. The development of novel formulations to enhance the stability of HNG peptides is a critical first step toward the therapeutic delivery of HNG in retinal degeneration models in vivo and for future clinical investigations to treat several age-related diseases such as AMD, Alzheimer’s disease, and diabetic retinopathy. To the best of our knowledge, this is the first study that accurately analyzes the stability features of HNG and identifies its fragments and their therapeutic potential using high-performance liquid chromatography (HPLC) and high-resolution mass spectrometry (HRMS). We also developed a stabilization formula (MO formulation) that significantly improves the HNG peptide stability when stored long term and at 37 °C.

## 2. Materials and Methods

### 2.1. Chemicals and Materials

The HNG peptide (Catalog No: AS-60887) was purchased from AnaSpec Inc. (Fremont, CA, US). Acetonitrile, HPLC water, LC-MS water, and formic acid were purchased from Fisher Scientific (Waltham, MA, USA). Analytical grade solvents were used in all experiments.

### 2.2. Physiochemical Properties

An ExPASy ProtParam bioinformatics software tool was used to determine structural prediction including the instability index value, grand average of hydropathy value (GRAVY), and theoretical isoelectric point (pI). The instability index represents the prediction of peptide instability. When a peptide’s instability index is less than 40, the peptide is classified as stable, and if it is higher than 40, the peptide is designated as unstable. The GRAVY method predicts peptide hydrophilicity and hydrophobicity. GRAVY’s positive values and negative values represent the hydrophobic and hydrophilic structures, respectively [26,27,28,29]. The ExPASy PeptideCutter bioinformatics software tool was utilized to predict potential cleave sites, cleaving enzymes, and chemicals in the HNG peptide [30]. Theoretical charge of HNG peptide over pH change was analyzed using the peptide analysis tool in the Thermo-Fisher Scientific website.

### 2.3. HNG Solution Preparation and Storage

For HPLC studies, HNG solutions were prepared at 125 μg/mL in HPLC water and at 112.5 μg/mL in the stabilization formula (MO formulation). The MO formulation, a proprietary solution, is a colorless liquid and includes organic acid (pH = 2.4–2.5) that has been found to be non-toxic to cells. We prepared duplicate HNG peptide solutions that were stored at two different temperatures (4 °C and 37 °C). For long-term stability analyses, HNG solutions were stored for 11 months at 4 °C. HNG solutions were filtered using 0.22 μm filters before HPLC analysis. The stability features of the stored HNG solutions were evaluated by HPLC at seven different time-points (6 h, 21 h, 33 h, day 3, day 7, day 14, and day 28). For HRMS studies, samples with a concentration of 30 μM HNG were prepared from the stored HPLC water and MO-formulation to analyze 11-month-old HNG products.

### 2.4. High-Performance Liquid Chromatography

The Agilent 1100 system with an Agilent Eclipse XDB-C8 5 μm, 4.6 × 150 mm HPLC column was used to achieve liquid chromatographic separation. HNG was monitored at a wavelength of 200 nm using a Diode Array Detector. Gradient elution was performed with solvent A (water with 0.05% trifluoroacetic acid) and solvent B (acetonitrile with 0.05% trifluoroacetic acid). The gradient started at 30% solvent B with a ramp to 60% solvent B in a period of 10 min. At 12 min, the gradients begin to return to 30% solvent B in 0.1 min. The column was equilibrated from 12.1 min to 20 min at 30% solvent B (Table S1). The flow rate was set to 1 mL/min, and 25 μL of the sample was injected into the column. The column temperature was set at 40 °C.

### 2.5. High-Resolution Mass Spectrometry

The Waters^®^ Acquity H-class ultra-performance liquid chromatography (UPLC) method was run on a Waters BEH C4 column 300 Å, 1.7 μm, 50 mm × 2.1 using 25 min gradient at 0.3 mL/min from 97% A to 97% B, where A is 0.1% formic acid in water and B is 100% ACN (Table S2). Mass spectrometric analysis was performed using a XEVO G2-XS Quadrupole Time-of-Flight (QTof) mass spectrometer equipped with StepWave ion optics (Waters Corp., MA, USA). The positive electrospray ionization mode was utilized. Measurements were conducted using an ion source desolvation temperature of 350 °C and a cone voltage of 40 V. Argon was utilized as damping gas in the Collision-Induced Dissociation (CID) experiments. A capillary transfer temperature of 300 °C and a spray voltage of 3.0 kV were used to accomplish ionization. A resolution of 30,000 Full Width at Half Maximum (FWHM) was used for a full scan experiment within a range of *m*/*z* 100–2000 in addition to 15,000 FWHM with an isolation window adjusted to *m*/*z* 2.0 for Parallel Reaction Monitoring (PRM) mode. The instrument was operated in MSE continuum mode, which alternates low-energy (6 V) and high-energy (40 V) scans every 0.5 sec. Leucine Enkephalin was used as a lock mass for nominal mass correction, and a CsNaI ladder was used for detector calibration.

Mass to Charge Calculation Formula for Dimerized Form HNG and their Fragments. The disulfide bridge causes a mass shift of −2 Da. The monoisotopic HNG molecular weight (Mw) is 2657.3.


$$Mass\;to\;Charge\;Ratio=\frac{Peptide\;1\;Mw+Peptide\;2\;Mw-Dimerization\;Mass\;Shift+Number\;of\;Protonation}{Charge\;State\;of\;Dimer}$$

Mass to Charge Calculation Formula for oxidized form HNG and their fragments. Oxidation causes a mass shift of +16 Da. The monoisotopic HNG molecular weight (Mw) is 2657.3.

$$Mass\;to\;Charge\;Ratio=\frac{Peptide\;\;Mw+Oxidation\;Mass+Number\;of\;Protonation}{Charge\;State\;of\;Oxidized\;Peptide}$$

### 2.6. Data Analysis

Collected data were analyzed using MassLynx (version 4.2, 2016) and BiopharmaLynx (version 4.0.27.10, 2015) software programs provided by the Waters Company (Milford, MA, USA).

## 3. Results

**Figure 1:**
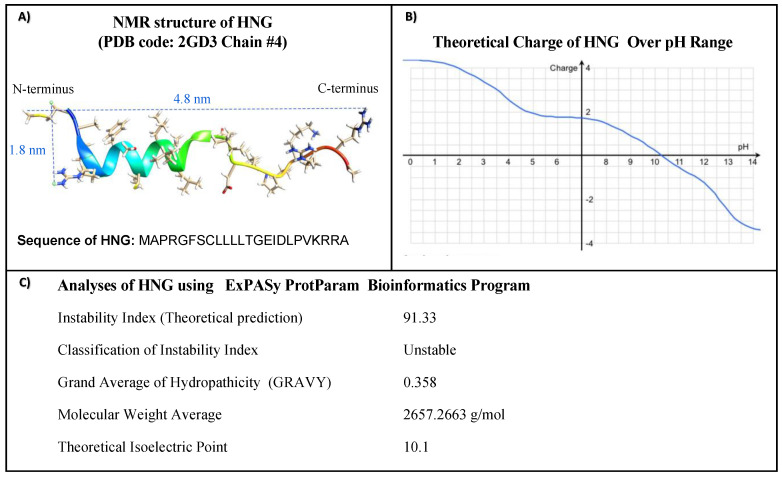
(**A**) The NMR structure of HNG peptide (PDB identifier 2GD3 Chain 8) is visualized using the UCSF Chimera program. The length and width of HNG are ~4 nm and ~1.5 nm, respectively. The sequence of full-length HNG is shown. (**B**) The diagram shows the net charge of HNG as a function of pH. (**C**) Physiochemical properties of HNG.

The molecular structure, molecular weights, charges, and amino acid sequences of HNG peptides were characterized using UCSF Chimera software and the ExPASy ProtParam bioinformatics software tool (Figure 1).

In Figure 1A, the predicted structure of HNG shows the proposed length and width as approximately 4.8 nm and approximately 1.8 nm, respectively. In Figure 1B, the isoelectric point is 10. 1, indicating it is a basic peptide. The net charge of HNG peptide at pH 7 is 1.9, indicating it is a soluble peptide in neutral water. In Figure 1C, HNG has an Instability Index of 91.33, suggesting it is an unstable peptide. The GRAVY value is 0.358, indicating a hydrophobic property.

We used the ExPASy PeptideCutter bioinformatics software to predict potential cleave sites, cleaving enzymes, and chemicals in the HNG peptide [30]. The functions, hydropathicity, name of cleaving enzymes/chemicals, and properties of each amino acid of HNG peptide are given in Table 1.

### 3.1. Short-Term Stability of HNG Peptide in Different Conditions

**Figure 2:**
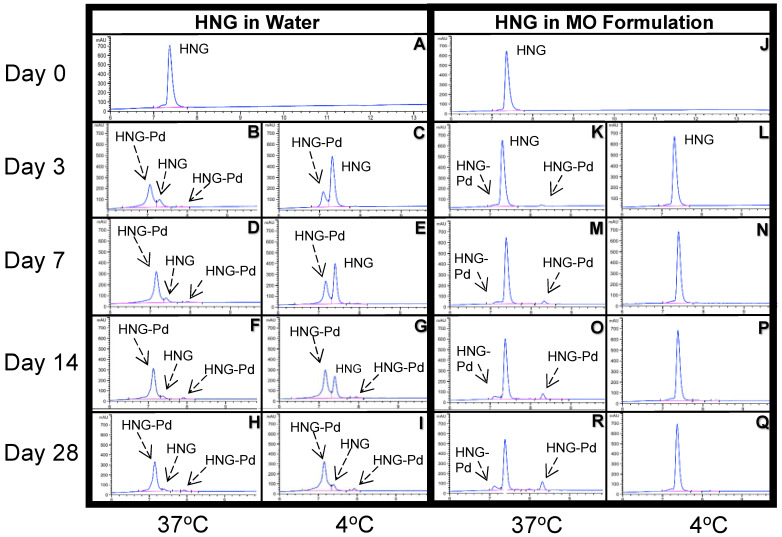
Ion chromatogram results of HNG in HPLC-grade water (Left Panel) and MO formulation (Right Panel). **LEFT PANEL: HNG in Water.** (**A**) Day 0 at RT; (**B**) 3rd day +37 °C; (**C**) 3rd day +4 °C; (**D**) 7th day +37 °C; (**E**) 7th day +4 °C. (**F**) 14th day +37 °C; (**G**) 14th day +4 °C; (**H**) 28th day +37 °C; (**I**) 28th day +4 °C. **RIGHT PANEL: HNG in MO formulation.** (**J**) Day 0 at RT; (**K**) 3rd day +37 °C; (**L**) 3rd day +4 °C; (**M**) 7th day +37 °C; (**N**) 7th day +4 °C; (**O**) 14th day +37 °C; (**P**) 14th day +4 °C; (**R**) 28th day +37 °C; (**Q**) 28th day +4 °C. RT, room temperature: HNG Humanin-G; Pd: Product.

We analyzed the stability of the HNG peptide stored in HPLC water (Figure 2A–I, Left Panel) and MO formulation (Figure 2J–Q, Right Panel) at 4 °C and 37 °C using HPLC at day 0, day 1, day 3, day 7, day 14, and day 28. The concentration of full-length HNG decreases over time, while the concentration of the HNG products (HNG-Pd) simultaneously increased (Figure 2A–R).

**Figure 3:**
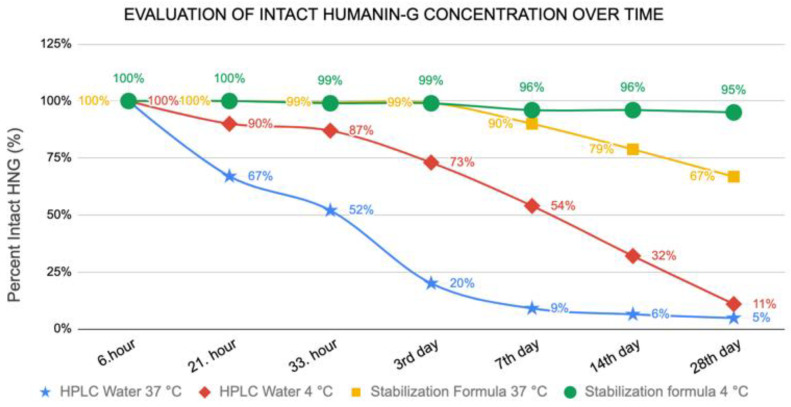
Stable HNG peptide concentration changes over time in HPLC water versus MO formulation at 4 °C and 37 °C.

Compared to the MO formulation, we found that the HNG peptide was sensitive to storage temperature and duration. At 21 h after storage, the full-length HNG peptide level in HPLC water stored at 4 °C was 90% compared to 67% at 37 °C (Figure 3). After 33 h of storage, nearly half of the full-length HNG peptides (52%) was found at 37 °C in HPLC water, indicating its half-life at body temperature (Figure 3). By day 28, the full-length HNG stored in HPLC water was further declined to concentrations of 11% at 4 °C and 5% at 37 °C (Figure 3).

When we evaluated the HNG stability stored in the MO formulation, remarkably, we found that the full-length HNG peptide remained stable up to 95% at 4 °C on day 28. Even at 37 °C, the full-length HNG peptide concentration was significantly higher when stored in the MO formulation compared to HPLC water (67% versus 11%, respectively) (Figure 3). These results show that the HNG peptide is highly unstable when stored in HPLC water, and the stability of the HNG peptide can be successfully improved when stored in our newly developed MO formulation.

**Figure 4:**
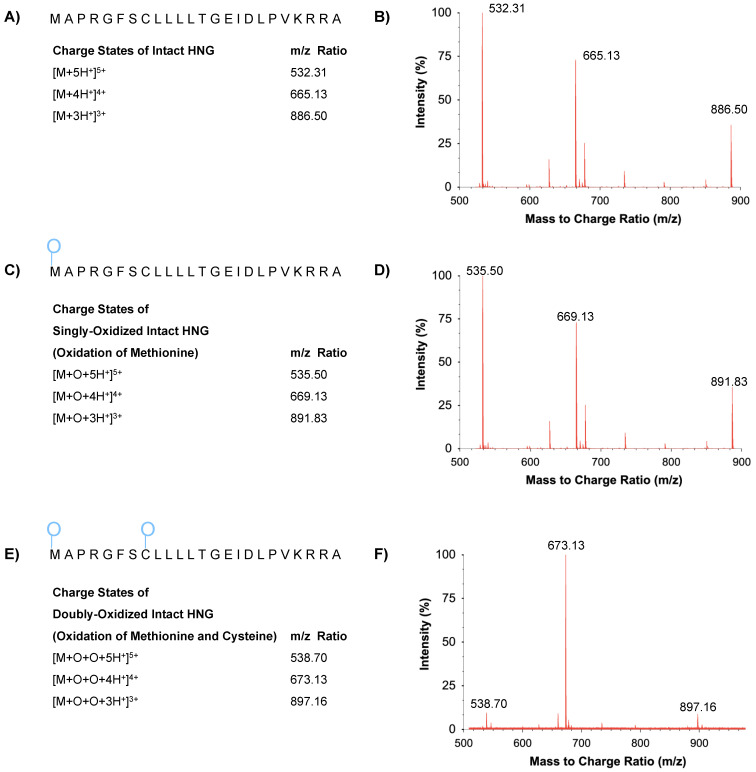
(**A**) Amino acid sequence and *m*/*z* ratio of the full-length HNG are represented. (**B**) Representative HRMS precursor ion mass spectra of HNG in PBS. (**C**) Amino acid sequence and *m*/*z* ratio of SO_X_-HNG are represented. (**D**) Representative HRMS precursor ion mass spectra of SO_X_-HNG in PBS. (**E**) Amino acid sequence and *m*/*z* ratio of DO_X_-HNG HNG are represented. (**F**) Representative HRMS precursor ion mass spectra of DO_X_-HNG in PBS.

**Figure 5:**
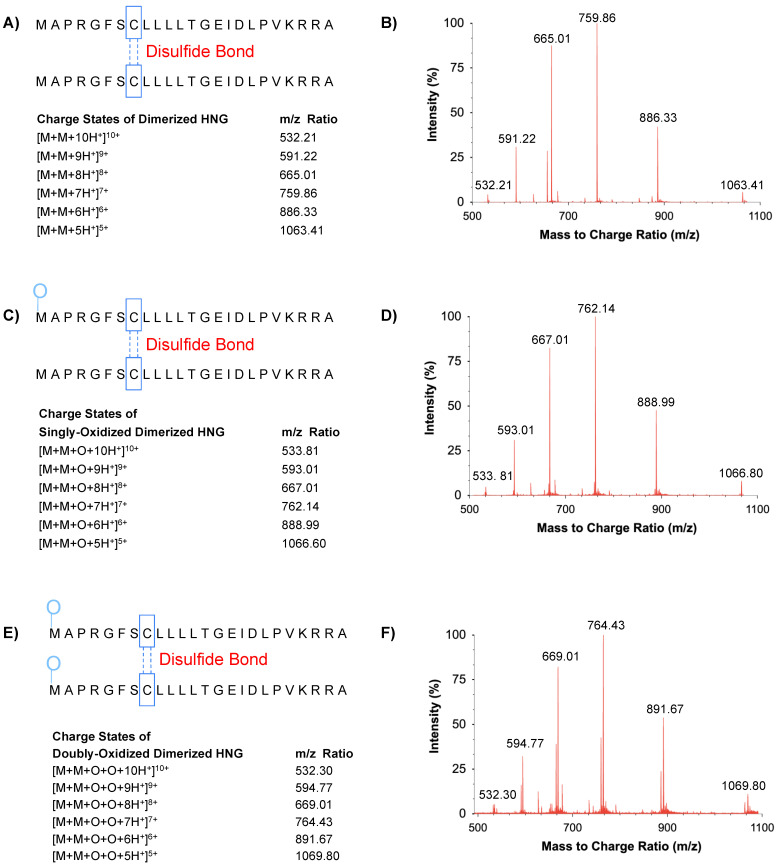
(**A**) Amino acid sequence and *m*/*z* ratio of DM-HNG are represented. (**B**) Representative HRMS precursor ion mass spectra of DM-HNG in PBS. (**C**) Amino acid sequence and *m*/*z* ratio of SO_X_-DM-HNG are represented. (**D**) Representative HRMS precursor ion mass spectra of SO_X_-DM-HNG in PBS. (**E**) Amino acid sequence and *m*/*z* ratio of DO_X_-DM-HNG are represented. (**F**) Representative HRMS precursor ion mass spectra of DO_X_-DM-HNG in PBS.

**Figure 6:**
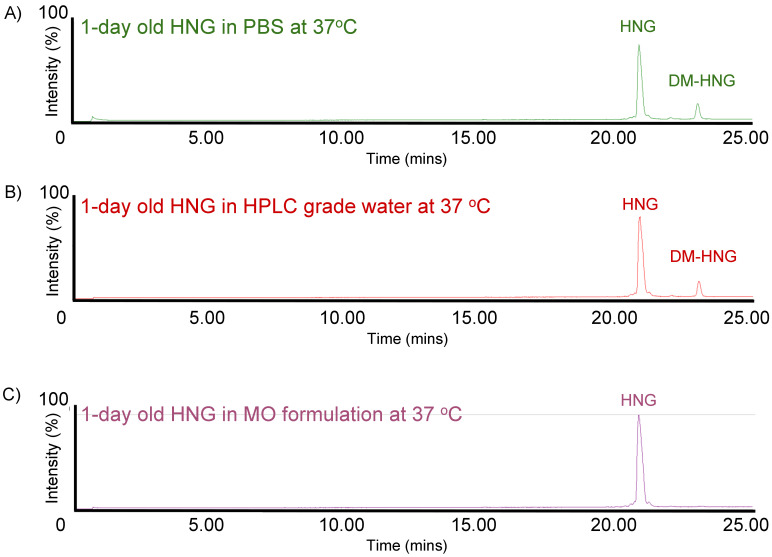
Ion chromatogram results of incubated HNG at 37 °C for 1 day in PBS (**A**), HPLC-grade water (**B**)**,** and MO formulation (**C**).

**Figure 7:**
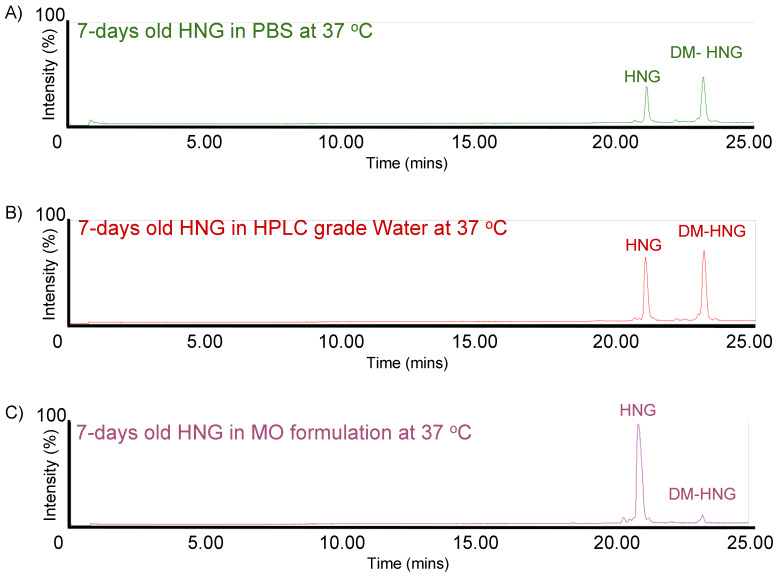
Ion chromatogram results of incubated HNG at 37 °C for 7 days in PBS (**A**), HPLC-grade water (**B**)**,** and MO formulation (**C**).

**Figure 8:**
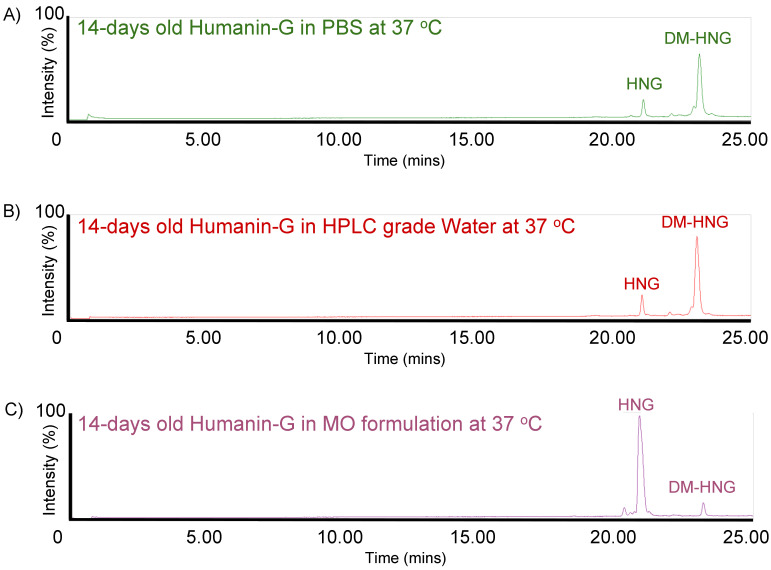
Ion chromatogram results of incubated HNG at 37 °C for 14 days in PBS (**A**), HPLC-grade water (**B**)**,** and MO formulation (**C**).

**Figure 9:**
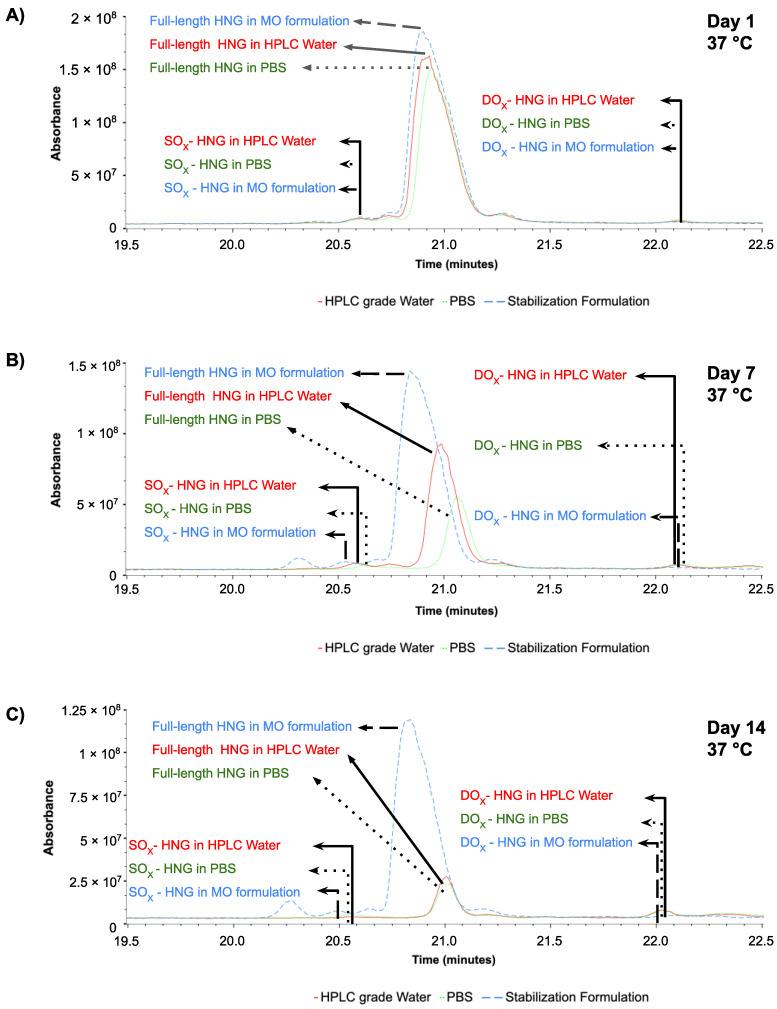
Overlay ion chromatogram (19 min–22.5 min) results of incubated HNG at 37 °C in PBS, HPLC-grade water and MO formulation at day 1 (**A**), at day 7 (**B**), and at day 14 (**C**).

**Figure 10:**
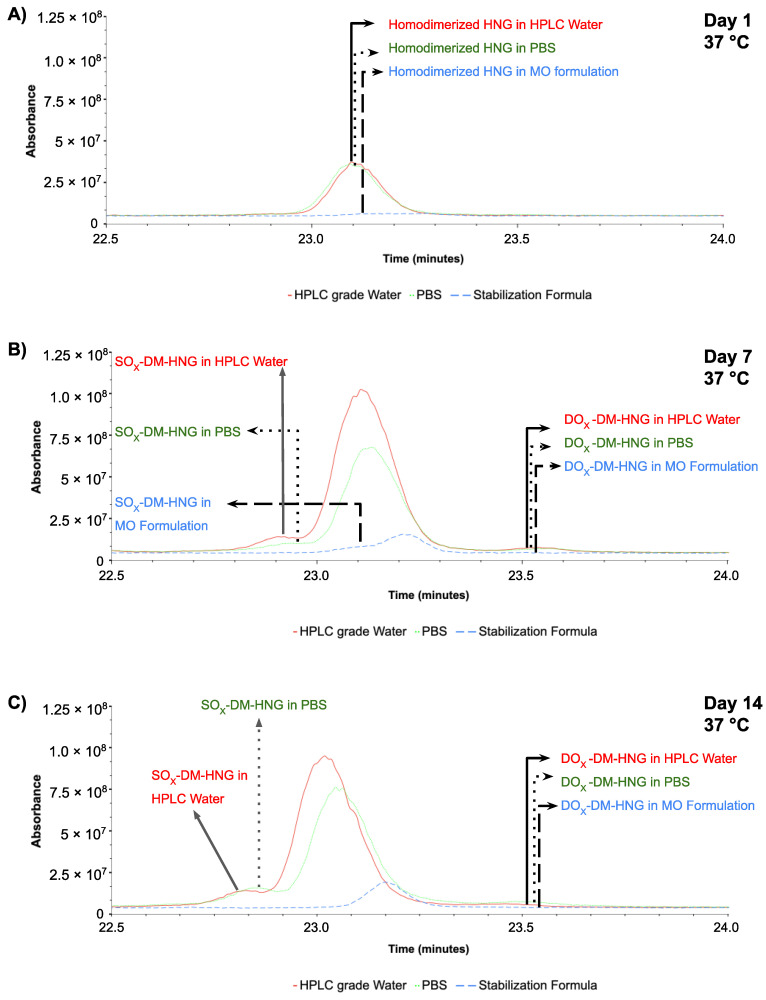
Overlay ion chromatogram (22.5 min–24 min) results of incubated HNG at 37 °C in PBS, HPLC-grade water and MO formulation at day 1 (**A**), at day 7 (**B**), at day 14 (**C**).

**Figure 11:**
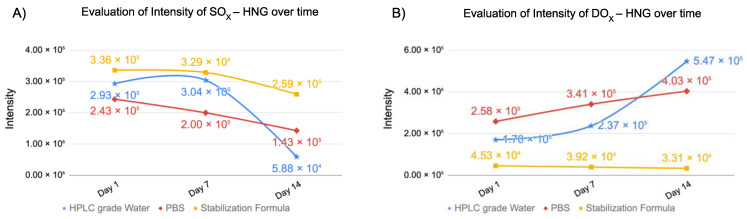
Evaluation of intensity changes of SO_x_-HNG (**A**), DOX-HNG (**B**) at day 1, 7 and 14.

**Figure 12:**
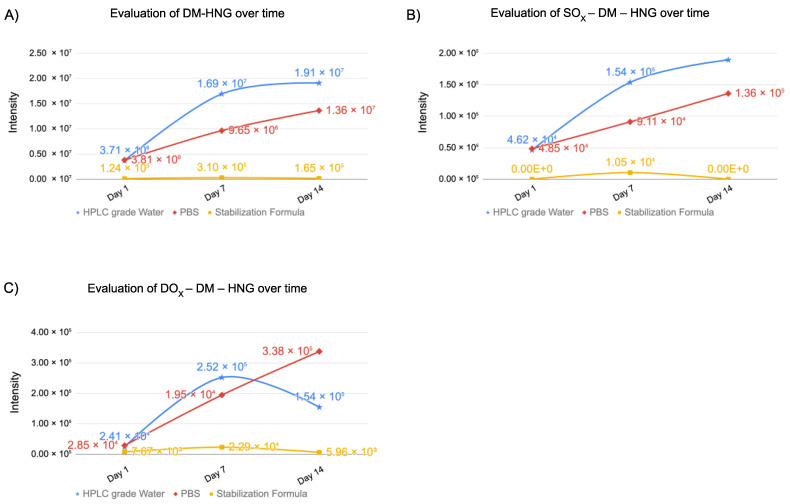
Evaluation of intensity changes of homodimerized form of HNG (**A**), SO_X_-DM-HNG (**B**), DO_X_-DM-HNG (**C**) at day 1, 7 and 14.

**Figure 13:**
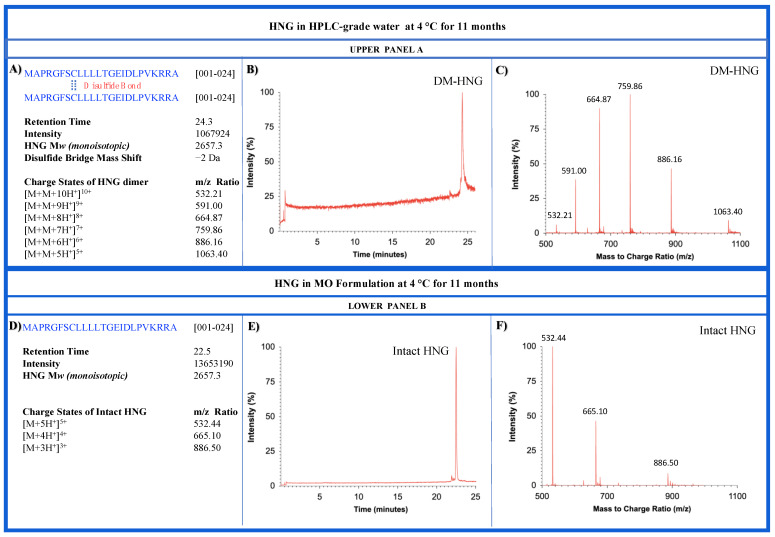
Measurements of HNG in HPLC-grade water (Upper Panel A) and MO formulation (Lower Panel B) at 4 °C for 11 months using HRMS. **UPPER PANEL A,** (**A**) Amino acid sequence and *m*/*z* ratio of dimerized form HNG is represented; (**B**,**C**) Representative ion chromatogram and HRMS product ion mass spectra of HNG in HPLC water, respectively. **LOWER PANEL B,** (**D**) Amino acid sequence and *m*/*z* ratio of HNG is represented; (**E**,**F**) Representative ion chromatogram and HRMS product ion mass spectra of HNG in MO formulation.

Overall, the stability of the full-length HNG peptide in HPLC water and MO formulation was measured using HPLC at 4 °C and 37 °C over a 28-day period, and the results showed that the full-length HNG peptide in HPLC-grade water is not stable at 4 °C and 37 °C. Based on the results of these HPLC studies, we saw indications, represented by the other peaks in the graph, of unidentified amino acid sequences of the HNG products. To identify the composition of HNG products that occurred in the three different solutions (HPLC-grade water, PBS, and MO formulation), we performed experiments using UPLC-HRMS. Collected UPLC-HRMS data were analyzed using the BiopharmaLynx program to identify the peptide sequence of each peak (Figure 4, Figure 5, Figure 6, Figure 7, Figure 8, Figure 9, Figure 10, Figure 11, Figure 12 and Figure 13, Tables S3 and S4).

### 3.2. Characterization of Full-Length HNG, Its Oxidized and Dimerized Forms

To characterize HNG products using UPLC-HRMS, we prepared a solution of HNG peptide in HPLC-grade water, PBS and MO formulation. Using UPLC-HRMS, the full-length HNG, singly oxidized full-length HNG (SO_x_-HNG), doubly oxidized full-length HNG (Dox-HNG), singly oxidized dimerized HNG (SO_x_-DM-HNG) and doubly oxidized dimerized HNG (DO_x_-DM-HNG) were identified.

The amino acid sequences and *m*/*z* ratio of full-length HNG, singly oxidized HNG, doubly oxidized HNG in PBS on day 14 at 37 °C are represented (Figure 4). In Figure 4A,B, multiple charged full-length HNG peptides (MAPRGFSCLLLLTGEIDLPVKRRA) were observed at *m/z* 532.31 (z = 5), 665.13 (z = 4), and 886.50 (z = 3). In Figure 4C,D, multiple charged singly oxidized (methionine) full-length HNG peptides (SO_x_-HNG) were observed at *m/z* 535.50 (z = 5), 669.13 (z = 4), and 891.83 (z = 3). In Figure 4E,F, multiple charged doubly oxidized (methionine and cysteine) full-length HNG peptides (Dox-HNG) were observed at *m/z* 538.70 (z = 5), 673.13 (z = 4), and 897.17 (z = 3).

In Figure 5, the amino acid sequences and *m*/*z* ratio of intact homodimerized (cysteine-cysteine disulfide bone) HNG (DM-HNG), singly oxidized homodimerized HNG, and doubly oxidized homodimerized HNG in PBS on day 14 at 37 °C are represented. In Figure 5A,B, multiple charged DM-HNG peptides were observed at 532.21 *m*/*z* (DM-HNG 1-24, z = 10), 591.22 *m*/*z* (DM-HNG 1-24, z = 9), 665.01 m/z (DM-HNG 1-24, z = 8), 759.86 *m*/*z* (DM-HNG 1-24, z = 7), 886.33 *m*/*z* (DM-HNG 1-24, z = 6), and 1063.41 *m*/*z* (DM-HNG 1-24, z = 5).

In Figure 5C,D, multiple charged homodimerized HNG with methionine oxidation (singly oxidized homodimerized HNG, SO_x_-DM-HNG) were observed at 533.81 *m*/*z* (z = 10), 593.01 *m*/*z* (z = 9), 667.01 *m*/*z* (z = 8), 762.14 *m*/*z* (z = 7), 888.99 *m*/*z* (z = 6), and 1066.80 *m*/*z* (z = 5).

In Figure 5E,F, multiple charged homodimerized HNG with methionine oxidized and cysteine disulfide bonds (doubly oxidized homodimerized HNG, DO_x_-DM-HNG) were observed at 532.30 *m*/*z* (z = 10), 594.77 *m*/*z* (z = 9), 669.01 *m*/*z* (z = 8), 764.43 *m*/*z* (z = 7), 891.67 *m*/*z* (z = 6), and 1069.80 *m*/*z* (z = 5).

HNG solutions were incubated at 37 °C and analyzed at day 1 (Figure 6), day 7 (Figure 7) and day 14 (Figure 8) using ultra-performance liquid chromatography coupled with high-resolution mass spectrometry (Waters^®^ Xevo G2-XS QTof). The presence of full-length HNG and DM-HNG in PBS, HPLC-grade water and MO formulation at the different time periods was analyzed (Figure 6, Figure 7 and Figure 8). The retention time frames of full-length HNG and DM-HNG ranged from 20.75 to 21.25 min and 22.50 to 23.25 min, respectively (Figure 6, Figure 7 and Figure 8).

Concentrations of full-length HNG peptide declined over time with a corresponding appearance of new peaks that increased over time (Figure 6, Figure 7 and Figure 8). These new peaks were identified as oxidized and/or dimerized HNG products. The DM-HNG was the dominant HNG-Pd at all time points. We found that the full-length HNG peptide had oxidized and dimerized at 37 °C in the PBS, the HPLC-grade water, and MO formulation at day 1 (Figure 6A–C), day 7 (Figure 7A–C) and day 14 (Figure 8A–C). The concentration of the DM-HNG simultaneously increased over time, while the HNG stored in the MO formulation remained mostly intact. (Figure 6, Figure 7 and Figure 8).

The full-length HNG, SO_x_-HNG and DO_x_-HNG were evaluated over time in PBS, HPLC-grade water and MO formulation. In Figure 9A, at day 1, SO_x_-HNG and DO_x_-HNG were detected in the HPLC-grade water as well as in the PBS and the MO formulation at 37 °C. The highest intensities of full-length HNG, and SO_x_-HNG were detected in the MO-formula, and next were those in the HPLC-grade water, with the lowest in the PBS. The highest intensity of DO_x_-HNG was detected in the PBS solution, while that in the HPLC-grade water was lower and that in the MO formulation was the lowest.

In Figure 9B, at day 7, higher intensities of SO_x_-HNG peptides and full-length HNG were detected in the MO formulation, next were those in the HPLC-grade water and the lowest were in the PBS at 37 °C. The highest intensity of DO_x_-HNG was detected in the PBS, that in the HPLC-grade water was lower and that in the MO formulation was lowest.

In Figure 9C, on day 14 at 37 °C, higher intensities of full-length HNG peptides were detected in the MO formulation, next were those in the HPLC-grade water and the lowest were in the PBS at 37 °C. Higher intensities of SO_x_-HNG peptides were detected in the MO formulation, next were those in the PBS and the lowest were in the HPLC-grade water. The highest intensity of DO_x_-HNG was detected in the HPLC-grade water, that in the PBS was lower and that in the MO formulation was the lowest.

The homodimerized form of HNG, SO_x_-DM-HNG and DO_x_-DM-HNG were evaluated over time in PBS, HPLC-grade water and MO formula at 37 °C. In Figure 10A, at day 1, a lower intensity of DM-HNG was detected in MO formula than HPLC-grade water and PBS.

In Figure 10B, on day 7 at 37 °C, higher intensities of SO_x_-DM-HNG peptides and DM-HNG were detected in the HPLC-grade water, next were those in the PBS and the lowest were in the MO formulation at 37 °C. The highest intensity of DO_x_-DM-HNG was detected in the HPLC-grade water, that in the PBS was lower and that in the MO formulation was lowest.

In Figure 10C, on day 14 at 37 °C, higher intensities of SO_x_-DM-HNG peptides and DM-HNG were detected in the HPLC-grade water, next were those in the PBS and the lowest were in the MO-formula at 37 °C. The highest intensity of DO_x_-DM-HNG was detected in the PBS, that in the HPLC-grade water was lower and that in the MO-formula was lowest.

In Figure 11A, intensities of SO_x_-HNG peptides were evaluated at 37 °C in various solutions at day 1, day 7 and day 14 using UPLC-HRMS. At days 1 and 7, higher intensity of SO_x_-HNG were detected in the MO formulation, next were those in the HPLC-grade water, with the lowest in the PBS. At day 14, higher intensities of SO_x_-HNG peptides were detected in the MO formulation, next were those in the PBS and the lowest were in the HPLC-grade water.

In Figure 11B, intensities of Dox-HNG peptides were evaluated at 37 °C in various solutions at day 1, day 7 and day 14 using UPLC-HRMS. At days 1 and 7, higher intensity of DO_x_-HNG was detected in the PBS solution, that in the HPLC-grade water was lower and that in the MO formulation was the lowest. At day 14, higher intensities of DO_x_-HNG peptides were detected in the HPLC-grade water, next were those in the PBS and the lowest were in the MO formulation.

In Figure 12A, intensities of DM-HNG peptides were evaluated at 37 °C in various solutions at day 1, day 7 and day 14 using UPLC-HRMS. At day 1, lower intensities of DM-HNG were detected in MO formulation than HPLC-grade water and PBS. At day 7, higher intensities of DM-HNG were detected in the HPLC-grade water, next were those in the PBS and the lowest were in the MO formulation at 37 °C. At day 14, a higher intensity of DM-HNG was detected in the HPLC-grade water, next was that in the PBS and the lowest was in the MO formulation at 37 °C.

In Figure 12B, intensities of SO_X_-DM-HNG were evaluated at 37 °C in various solutions at day 1, day 7 and day 14 using UPLC-HRMS. At day 1, SO_X_-DM-HNG was detected in HPLC-grade water and PBS and not detected in MO formulation. At day 7, a higher intensity of SO_x_-DM-HNG was detected in the HPLC-grade water, next was that in the PBS and the lowest was in the MO formulation at 37 °C. At day 14, higher intensities of SO_x_-DM-HNG were detected in the HPLC-grade water, next were those in the PBS and the lowest were in the MO formulation at 37 °C.

In Figure 12C, intensities of DO_X_-DM-HNG were evaluated at 37 °C in various solutions at day 1, day 7 and day 14 using UPLC-HRMS. At day 1, a lower intensity of DO_X_-DM-HNG was detected in MO formulation than HPLC-grade water and PBS. At day 7, higher intensities of DO_x_-DM-HNG peptides were detected in the HPLC-grade water, next were those in the PBS and the lowest were in the MO formulation at 37 °C. At day 14, the highest intensity of DO_x_-DM-HNG was detected in the PBS, that in the HPLC-grade water was lower and that in the MO formulation was the lowest.

### 3.3. Long-Term Stability of HNG Peptide in Different Conditions

We evaluated the long-term stability of HNG peptide in the HPLC water and MO formulation stored at 4 °C for 11 months. Collected HRMS data were analyzed using the BiopharmaLynx program to identify peptide sequences (Tables S3 and S4). Identified peptide sequences and HRMS data collected from 11 months old HNG in HPLC water (Table S3) and MO formulation (Table S4) show the full-length HNG peptide, its fragments, and dimerized forms. Mass spectrometry analysis showed that the HNG peptide in HPLC water degraded into multiple fragments (Figure 13B), while in MO formulation, HNG remained mostly intact (Figure 13E). The retention time frames of 11-month-old DM-HNG in HPLC water ranged from 24.2 to 24.6 min (Figure 13A,B, Upper Panel A) and 11-month-old HNG in MO formulation ranged from 22.4 to 22.6 min (Figure 13D,E, Lower Panel B). The 11-month-old HNG peptides in HPLC water showed multiple charged states 532.2 *m/z* (homodimerized-HNG 1-24, z = 10), 591.0 *m/z* (DM-HNG 1-24, z = 9), 664.9 *m*/*z* (DM-HNG 1-24, z = 8), 759.9 *m/z* (DM-HNG 1-24, z = 7), 886.2 *m*/*z* (DM-HNG 1-24, z = 6), and 1063.4 *m*/*z* (DM-HNG 1-24, z = 5) as shown in Figure 13C, Upper Panel A. Many fewer multiple charged intact molecules of 11-month-old HNG peptides in MO formulation were observed at *m/z* 532.3 (HNG 1-24, z = 5), 665.1 (HNG 1-24, z = 4), and 886.5 (HNG 1-24, z = 3) (Figure 13F, Lower Panel B).

As seen in Figure 13, the HNG peptides without a stabilizing formulation were oxidized and dimerized continuously in the HPLC-grade water while the peptide oxidation and dimerization were much slower in the MO formulation. The HNG peptides were stored in the HPLC water (Table S3) and MO formulation (Table S4) over 11 months, and many dimers and oxidized products were identified in both solutions. However, the highest intensities of full-length HNG were found in MO formulation (Table S4), suggesting the potential of the MO formulation for general use in enhancing peptide stability and preventing peptide oxidation.

In summary, our results show that the full-length HNG peptide (24 amino acids) is highly susceptible to chemical modification when placed in HPLC water and PBS at either 4 °C or 37 °C. For example, when placed in HPLC water for 28 days at 4 °C, less than 11% was found in the full-length HNG peptide form (Figure 3), but the HNG peptide was stabilized when placed in the MO formulation (67% and 95% remained full-length HNG at 37 °C or 4 °C, respectively). Using UPLC-HRMS, the full-length HNG was found in both the singly oxidized and doubly oxidized forms (Figure 4); the ionized mass spectra of the SO_X_-HNG and DO_X_-HNG showed multiple charged states (Figure 4). The ion chromatography of the full-length HNG incubated for 1 to 14 days in PBS, HPLC-grade water or the MO formulations showed increasing loss in the full-length HNG in the PBS and HPLC-grade water, but surprisingly, the full-length HNG in the MO formulation remains mostly stable (Figure 6). Finally, when the full-length HNG was stored for 11 months in HPLC-grade water at 4 °C, we found full-length HNG, along with dimerization and degradation products of HNG, which included three different HNG fragments and four different dimerized forms of HNG fragments (Table S3). When the full-length HNG was stored for 11 months in the MO formulation at 4 °C, there were the full-length HNG, SO_X_-HNG, DO_X_-HNG and degradation products of HNG, which includes 28 different HNG fragments and 64 dimerized forms of HNG fragments (Table S4). These data demonstrate that the full-length HNG is fragmented and modified chemically in HPLC-grade water and the MO formulation. Future studies will investigate the biological features of the HNG fragments, the dimerized HNG and oxidized HNG, since these forms may have signaling functions, reflecting increased mitochondrial DNA damage and/or perhaps a positive, rescuing effect for damaged cells.

## 4. Discussion

Despite the promising results that demonstrated the key cellular protective role of HNG, the in vitro and in vivo stability and half-life properties of HNG peptide have not been well studied, and the majority of the reported studies investigating the role of HNG were mostly limited to in vitro cell cultures. Therefore, determining and enhancing the molecular stability properties of HNG is essential to better translate to proper in vivo therapeutic studies. Understanding the stability properties of full-length HNG peptide can help us more accurately determine the dosing and frequency for HNG administration in in vivo animal studies and for possible future clinical studies investigating its role in physiology and disease.

Water and moisture have many effects on peptide degradation [31]. A 28-amino acid Vasoactive Intestinal Peptide, a 29-amino acid peptide Glucagon, vaso-active intestinal peptide and a tricyclic glycopeptide Vancomycin are unstable in aqueous solutions [32,33,34,35]. Knoop et al. have reported the instability of the MOTS-c peptide, another mitochondria-derived peptide, in the human plasma [36]. Consistent with those findings, our results showed that HNG in HPLC water is unstable at 37°C, reaching 50% concentration at approximately 33 h. When stored in HPLC water at 4°C, then 54% of the HNG peptide remained stable at day 7, indicating peptide instability even in cold storage conditions in HPLC water. Therefore, researchers investigating the effects of the HNG peptide should consider this newly identified short half-life when determining treatment doses and frequency in cell cultures or in vivo administration to the systemic circulation.

To overcome the low stability issue of the HNG peptide, we developed a special solution (MO formulation) to improve the stability of the molecule. The MO formulation has an acidic property to stabilize the HNG peptide structure. Our proprietary solution demonstrated significant efficiency resulting in 95% full-length HNG peptides after 28 days of storage at 4 °C. Moreover, the MO formulation could provide a 95% stable HNG peptide concentration for up to 7 days at 37 °C. Hence, the MO formulation may significantly improve the efficacy of HNG treatment, and it could reduce administration frequency and costs as well. The increased stability with the MO formulation may provide further processing opportunities such as infusion of HNG and/or potentially other MDPs such as small humanin-like peptides into microspheres for various applications.

Oxidative mechanisms play critical roles in aging and age-related diseases such as ischemia, atherosclerosis, Alzheimer’s disease, cataracts, and AMD [37,38,39]. Peptide oxidation decreases enzymatic activity, accumulates with age, and is related to numerous diseases [38]. Cysteine, methionine, histidine, and tryptophan amino acids are most susceptible to oxidation [38]. Oxidation causes a mass shift of +16 Da. HNG includes methionine and cysteine amino acids, which are susceptible to oxidation. Cysteine oxidation in HNG is responsible for the dimerization of HNG fragments via disulfide bridges. The disulfide bridge causes a mass shift of −2 Da and produces stable, covalently bonded dimers. The high-resolution tandem mass spectrometer provides highly sensitive and accurate results that can identify oxidation sites and disulfide bridges in peptides and dimerized peptides.

Finally, there is a lack of knowledge regarding the degradation products of HNG peptide and their oxidized and dimerized forms. Our study demonstrates that HNG fragments formed homodimers and heterodimers via disulfide bridge interactions (Tables S3 and S4). In the long-term study, our results show that dimerization provides increased stability for the intact HNG and its fragments (Figure 13D,F, Lower Panel B). Consistent with the other studies, several proteins have been shown to increase stability and have functions in dimerized forms, such as human IgG antibody [40], HLA-G dimers on cell surfaces [41], human superoxide dismutase enzymes [42], and glial cell line-derived neurotrophic factor [43]. Disulfide bonds contribute to the structure, functionality stability, and dimerization of peptides and proteins [40,41,42,43].

## 5. Conclusions

For the first time, the short- and long-term stability properties of HNG peptide and its oxidation and degradation products have been analyzed in detail using advanced HPLC and HRMS technologies. Our findings may provide insight for understanding key features in the HNG peptide sequence that define its stability via disulfide bonds. It is currently unknown whether dimerized HNG fragments possess any biological activity. Additionally, we have identified various HNG fragments that may possess different cellular functionalities and/or receptor activities. Future studies will investigate whether there are any such biological functions of these HNG fragments. We also developed a new chemical solution that significantly improves the stability of the HNG peptide in both 4 °C and 37 °C media conditions for up to 28 days. Our results may help researchers design better in vitro and in vivo experimental parameters to further understand the critical role of Humanin and HNG in physiological conditions and human diseases.

## Figures and Tables

**Table 1:** Functions, hydropathicity, name of cleaving enzymes/chemicals and properties of each amino acids of HNG peptide.

Pos.	Amino Acid	Function	Hydropathicity	Amino Acid Side Chain Properties	Name of Cleaving Enzymes/Chemicals (Theoretical)
1	M	Neuroprotection	1.900	Hydrophobic	Chymotrypsin, Cyanogen Bromide
2	A	Neuroprotection	1.800	Hydrophobic	Proteinase K
**3**	P*	**Neuroprotection**	−1.600	Hydrophobic	N/A
**4**	R*	**Neuroprotection**	−4.500	Positive Charged (Basic)	Trypsin, Arg-C proteinase, Clostridiopeptidase B
**5**	G*	**N/D**	−0.400	Hydrophobic	Pepsin, Thermolysin
**6**	F*	**IGFBP-3 binding, Beta-Amyloid Binding**	2.800	Hydrophobic	Chymotrypsin, Proteinase K
**7**	S*	**Beta-Amyloid Binding, Beta-Amyloid Protection, Dimerization**	−0.800	Polar	2-nitro-5-thiocyanobenzoic acid
**8**	C*	**Neuroprotection, BAX, BAD, end tBID binding, Disulfide Bond for dimerization**	2.500	Polar	Pepsin, Thermolysin
**9**	L*	**Neuroprotection, Secretion, Dimerization**	3.800	Hydrophobic	Proteinase K, Pepsin, Thermolysin, Chymotrypsin
**10**	L*	**Secretion**	3.800	Hydrophobic	Proteinase K, Pepsin, Thermolysin, Chymotrypsin
**11**	L*	**Secretion**	3.800	Hydrophobic	Proteinase K, Pepsin, Thermolysin, Chymotrypsin
**12**	L*	**Neuroprotection**	3.800	Hydrophobic	Proteinase K, Pepsin, Chymotrypsin
**13**	T*	**Neuroprotection**	−0.700	Polar	Proteinase K,
**14**	G*	**Neuroprotection**	−0.400	Hydrophobic	Asp-N Endopeptidase
**15**	E*	**N/D**	−3.500	Negative Charged (Acidic)	Proteinase K, Glutamyl endopeptidase, Staphylococcal peptidase I
**16**	I*	**N/D**	4.500	Hydrophobic	Proteinase K, Asp-N Endopeptidase
**17**	D*	**N/D**	−3.500	Negative Charged (Acidic)	Formic acid
**18**	L*	**N/D**	3.800	Hydrophobic	Proteinase K, Pepsin
**19**	P*	**Beta-Amyloid Protection, Secretion**	−1.600	Hydrophobic	Thermolysin
**20**	V*	**Secretion**	4.200	Hydrophobic	Proteinase K, Peptidyl-Lys metalloendopeptidase
21	K	IGFBP-3 binding	−3.900	Positive Charged (Basic)	Lysyl endopeptidase, Trypsin
22	R	N/D	−4.500	Positive Charged (Basic)	Arg-C proteinase, Clostridiopeptidase B, Trypsin
23	R	N/D	−4.500	Positive Charged (Basic)	Thermolysin, Arg-C proteinase, Clostridiopeptidase B, Trypsin
24	A	N/D	1.800	Hydrophobic	N/A

* Neuroprotection core domains are shown in bold font. Pos.; Position, N/D; Not Determined, N/A: Not Available.

## Data Availability

Not applicable.

## Supplementary Materials

The following supporting information can be downloaded at: https://www.mdpi.com/article/10.3390/biom13030515/s1, Table S1: Represent HPLC gradient run for stability assay. Table S2: Represent UPLC gradient for identification of Humanin-G product. Table S3: Amino acid sequences and mass spectrometric analytical properties of HNG in HPLC water at 11 months. Dimerized HNG and dimerized HNG fragments are ordered by intensity (counts). Table S4: Amino acid sequences and mass spectrometric analytical properties of HNG in MO formulation at 11 months. Dimerized HNG and dimerized HNG fragments are ordered by intensity (counts).
