# Supplementary material for: Stability Determination of Intact Humanin-G with Characterizations of Oxidation and Dimerization Patterns
Source: Biomolecules. 2023 Mar 11;13(3):515. doi: 10.3390/biom13030515 (PMC10046509; doi:10.3390/biom13030515)
Supplement: Supplementary file 1 [file biomolecules-13-00515-s001.zip › Table S1.pdf]

Table S1. Represent HPLC gradient run for stability assay.

Time (min)	Flow (mL/min)	% of Solution A	% of Solution B
0	1.00	70.0	30.0
10	1.00	40.0	60.0
12.00	1.00	40.0	60.0
12.10	1.00	30.0	30.0
21.00	1.00	30.0	30.0

* Min; Minutes, mL/min; Milliliters per minute.
