# Supplementary material for: Stability Determination of Intact Humanin-G with Characterizations of Oxidation and Dimerization Patterns
Source: Biomolecules. 2023 Mar 11;13(3):515. doi: 10.3390/biom13030515 (PMC10046509; doi:10.3390/biom13030515)
Supplement: Supplementary file 1 [file biomolecules-13-00515-s001.zip › Table S2.pdf]

Table S2. Represent UPLC gradient for identification of Humanin-G product.

**Time**	**Flow (mL/min)**	**% of Solution A**	**% of Solution B**	**Curve**
Initial	0.300	97.0	3.0	Initial
1.00	0.300	97.0	3.0	6
25.00	0.300	73.0	27.0	6
27.00	0.300	10.0	90.0	6
27.5	0.300	10	90	6
29.00	0.300	97.0	3.0	6
30.00	0.050	97.0	3.0	6

* Min; Minutes, mL/min; Milliliters per minute.
