# Supplementary material for: Stability Determination of Intact Humanin-G with Characterizations of Oxidation and Dimerization Patterns
Source: Biomolecules. 2023 Mar 11;13(3):515. doi: 10.3390/biom13030515 (PMC10046509; doi:10.3390/biom13030515)
Supplement: Supplementary file 1 [file biomolecules-13-00515-s001.zip › Table S3.pdf]

Table S3. Amino acid sequences and mass spectrometric analytical properties of HNG in HPLC water at 11 months. Dimerized HNG and dimerized HNG fragments are ordered by intensity (counts).

Peptide	Label	Modifier	RT (Min)	Control Charge State	Control m/z	Control Mass (Da)	Control Intensity (Counts)	Control b/y Found	Control b/y List
**MAPRGFSCLLLLTGEIDLPVKRRA= MAPRGFSCLLLLTGEIDLPVKRRA**	1:F001-024-2:F001-024	N.D.	24.3	7	759.4249	5308.9189	1067924	30	1/b6;1/b7;1/y2;1/y4;1/y6;1/y8;1/y9;1/y10;1/y14;1/y15;1/y24-2/y22;1/y24-2/b12;1/y24-2/b23;1/y24-2/b17;1/y24-2/b10;1/y24-2/b15;1/y24-2/b9;1/y24-2/b11;1/y24-2/b13;1/y24-2/y21;2/b2;2/b3;2/b4;2/b5;2/y5;2/y7;2/y11;2/y12;2/y13;2/y16
**MAPRGFSCLLLLTGEIDLPVKRRA**	1:F001-024	N.D.	22.6	4	664.8752	2655.469	29714	10	b8;b9;b10;b11;y7;y9;y11;y12;y13;y14
**TGEIDLPVKRRA**	1:F013-024	N.D.	24.3	2	677.8915	1353.7671	12655	12	b2;b3;b4;b5;b6;b10;b11;y2;y4;y5;y6;y10
**FSCL=GFSCLLLLTGEIDLPVKRRA**	1:F005-024-2:F006-009	N.D.	24.3	3	889.657	2665.9473	11790	12	1/y4-2/b10;1/y4-2/b7;1/y17-2/y4;2/y2;2/y4;2/y5;2/y6;2/y10;2/y12;2/y14;2/y15;2/y16
**GFSCLLLLTGEIDLP=MAPRGFSCLL**	1:F001-010-2:F005-019	N.D.	24.3	3	894.813	2681.4153	11469	9	1/y2;1/y10;1/y11;2/b2;2/b3;2/b4;2/b5;2/b6;2/b7
**APRGFSC=MAPRGFSCLLLLTGEIDL**	1:F001-018-2:F002-008	N.D.	24.3	3	894.9783	2681.9111	10492	12	1/b4;1/b6;1/y7-2/b12;1/y7-2/b11;1/y18-2/y6;2/b2;2/b3;2/b4;2/b5;2/b6;2/b7;2/y8
**GEIDLPVKRRA**	1:F014-024	N.D.	24.3	2	627.3692	1252.7225	8465	10	b2;b3;b4;b5;b7;b10;y2;y4;y5;y10
**RGFSCLL=CLLLLT**	1:F004-010-2:F008-013	N.D.	24.3	2	734.4342	1466.8525	5275	7	1/y6-2/b6;1/y6-2/y4;1/y6-2/b5;1/y6-2/y6;1/y7-2/b1;1/y7-2/b5;1/y7-2/b4
**LTGEIDLPVKRRA**	1:F012-024	N.D.	24.3	2	734.4342	1466.8525	5275	10	b2;b4;b5;b6;b7;b12;y2;y4;y5;y12

The symbol "=" represents the dimerization via disulfide bonds between two peptide sequences. The first row is the dimerized HNG peptide while the remaining sequences are the dimerized HNG fragments. N.D.; Not detected. RT; Retention time, Min; Minutes.
