# Supplementary material for: Stability Determination of Intact Humanin-G with Characterizations of Oxidation and Dimerization Patterns
Source: Biomolecules. 2023 Mar 11;13(3):515. doi: 10.3390/biom13030515 (PMC10046509; doi:10.3390/biom13030515)
Supplement: Supplementary file 1 [file biomolecules-13-00515-s001.zip › Table S4.pdf]

Table S4. Amino acid sequences and mass spectrometric analytical properties of HNG in MO formulation at 11 months. Dimerized HNG and dimerized HNG fragments are ordered by intensity (counts).

Peptide	Label	Modifier	RT (Min)	Control Charge State	Control m/z	Control Mass (Da)	Control Intensity (Counts)	Control b/y Found	Control b/y List
**MAPRGFSCLLLLTGEIDLPVKRRA**	1:F001-024	N.D.	22.5	4	664.8777	2655.479	13653190	36	b2;b3;b4;b5;b6;b7;b8;b9;b10;b11;b12;b13;b14;b15;b16;b17;b18;b23;y2;y3;y4;y5;y6;y7;y8;y9;y10;y11;y12;y13;y14;y15;y17;y18;y22;y23
**MAPRGFSCLLLLTG=CLLLLTGEIDL**	1:F001-014-2:F008-018	N.D.	22.5	3	893.4903	2677.447	861882	24	1/y2;1/y3;1/y4;1/y6;1/y7;1/y7-2/y11;1/y8;1/y11-2/b12;1/y11-2/b13;1/y11-2/y13;1/y11-2/y10;1/y11-2/y9;1/y11-2/y11;1/y14-2/b1;2/b2;2/b3;2/b4;2/b5;2/b6;2/b7;2/y2;2/y3;2/y5;2/y6
**TGEIDLPVKRRA**	1:F013-024	N.D.	22.5	2	677.8936	1353.7712	538818	17	b2;b3;b4;b5;b6;b9;b10;b11;y2;y3;y4;y5;y6;y7;y8;y9;y10
**SCLLLLTGEIDLPVK=CLLLLTGEID**	1:F007-021-2:F008-017	N.D.	22.5	3	900.8174	2699.4285	406213	19	1/b8-2/y15;1/y2;1/y3;1/y6;1/y7;1/y8;1/y10-2/b12;1/y10-2/b2;1/y10-2/b6;1/y10-2/b8;1/y15-2/b2;1/y15-2/b9;1/y15-2/b3;2/y2;2/y3;2/y6;2/y9;2/y10;2/y12
**MAPRGFSCLLLLTGEID=CLLLLTGE**	1:F001-017-2:F008-015	N.D.	22.5	4	674.3619	2693.4158	381001	20	1/b7-2/y17;1/y2;1/y3;1/y4;1/y6;1/y8-2/y16;1/y8-2/b8;1/y8-2/b15;1/y15-2/y8;2/b2;2/b3;2/b4;2/b5;2/b6;2/b7;2/y2;2/y3;2/y6;2/y7;2/y8
**GEIDLPVKRRA**	1:F014-024	N.D.	22.5	2	627.3705	1252.7252	326385	16	b2;b3;b4;b5;b6;b7;b9;b10;y2;y3;y4;y5;y6;y8;y9;y10
**MAPRGFSCLLLLTGEIDLPVKRRA**	1:F001-024	N.D.	21.9	4	664.8751	2655.4688	282660	25	b2;b3;b4;b5;b7;b8;b9;b10;b11;b12;b13;b17;y2;y6;y7;y8;y9;y10;y11;y12;y13;y14;y15;y22;y23
**LTGEIDLPVKRRA**	1:F012-024	N.D.	22.5	2	734.4359	1466.856	223774	16	b2;b3;b4;b6;b7;b11;b12;y2;y3;y4;y5;y6;y8;y9;y10;y12
**FSCLLLL=FSCLLLLTGEIDLPVKR**	1:F006-012-2:F006-022	N.D.	22.5	3	908.1447	2721.4102	205875	16	1/b2;1/y2;1/y4;1/y7-2/b4;1/y7-2/b8;1/y7-2/b6;1/y7-2/b3;1/y7-2/b9;1/y7-2/b7;2/y1;2/y2;2/y3;2/y5;2/y9;2/y11;2/y14
**MAPRGFSCLLLLTGEIDLPVKRRA**	1:F001-024*	Oxidation	22.1	4	668.8735	2671.4622	169399	19	b6*;b7*;b8*;b9*;b10*;b11*;b12*;b13*;y

		M(1)							2;y6;y7;y8;y9;y10;y11;y12;y13;y14;y15
**PRGFSCLLLLTG=CLLLLTGEIDLPV**	1:F003-014-2:F008-020	N.D.	22.1	4	668.8735	2671.4622	169399	5	1/y2;1/y3;1/y11;2/b2;2/b4
**APRGFSCLL**	1:F002-010	N.D.	22.5	1	963.4548	962.4469	147884	9	b2;b3;b4;b6;b7;b8;y2;y3;y8
**APRGFSCLLL**	1:F002-011	N.D.	22.5	1	1076.5399	1075.532	140315	12	b2;b3;b4;b6;b7;b8;b9;y2;y5;y6;y7;y9
**GFSC=PRGFSC**	1:F003-008-2:F005-008	N.D.	22.5	1	1076.5399	1075.532	140315	6	1/b2;1/b3;2/b2;2/b3;2/b4;2/b5
**LLTGEIDLPVKRRA**	1:F011-024	N.D.	22.5	2	790.9772	1579.9386	85221	12	b2;b4;b6;b7;b11;b13;y2;y3;y4;y5;y8;y12
**APRGFSCL**	1:F002-009	N.D.	22.5	1	850.3707	849.3628	84132	9	b2;b3;b4;b6;b7;y2;y3;y5;y7
**MAPRGFSCLLLLTGEIDLPV=CLLLL**	1:F001-020-2:F008-012	N.D.	22.5	3	906.1381	2715.3904	81223	17	1/b18-2/y5;1/y2;1/y4;1/y5-2/b9;1/y5-2/b11;1/y19-2/y5;2/b2;2/b3;2/b4;2/b5;2/b6;2/b7;2/y2;2/y7;2/y10;2/y11;2/y12
**APRGFSC=FSCLLLLTGEIDLPVKRR**	1:F002-008-2:F006-023	N.D.	22.5	3	936.4689	2806.3828	80813	26	1/b2;1/b3;1/b4;1/b6;1/y7-2/b11;1/y7-2/b9;1/y7-2/y16;1/y7-2/b10;1/y7-2/b16;1/y7-2/b6;1/y18-2/y2;2/b2;2/y1;2/y2;2/y3;2/y4;2/y5;2/y6;2/y7;2/y8;2/y9;2/y10;2/y11;2/y12;2/y13;2/y14
**RGFSCLLLLTGEI=PRGFSCLLLLT**	1:F003-013-2:F004-016	N.D.	22.5	5	528.4999	2637.4597	79623	18	1/b2;1/b3;1/b4;1/b5;1/y1;1/y2;1/y3;1/y4;1/y5;1/y11-2/b7;1/y13-2/b10;2/b1;2/b2;2/b3;2/b4;2/y3;2/y4;2/y6
**APRGFSCLLLL**	1:F002-012	N.D.	22.5	2	595.3155	1188.6151	72797	12	b2;b3;b4;b6;b7;b8;b9;b10;y2;y4;y5;y10
**RGFSCLLL=CLLLLTGEIDLPVKRR**	1:F004-011-2:F008-023	N.D.	22.5	3	915.4716	2743.3909	67423	24	1/b7-2/y8;1/y1;1/y2;1/y3;1/y4;1/y5;1/y6;1/y7;1/y8-2/b3;1/y8-2/b2;1/y8-2/b5;1/y8;1/y8-2/b10;1/y9;1/y10;1/y11;1/y12;1/y13;1/y14;2/b1;2/b2;2/b3;2/b4;2/y2
**FSCLLL=FSCLLLLTGEIDLPVKRR**	1:F006-011-2:F006-023	N.D.	22.5	3	922.4716	2764.3909	53817	25	1/b2;1/y2;1/y6-2/b16;1/y6-2/b14;1/y6-2/b5;1/y6-2/b7;1/y6-2/b8;1/y6-2/b4;1/y6-2/b9;1/y6-2/b3;1/y6-2/b12;1/y18-2/b5;2/y1;2/y2;2/y3;2/y4;2/y5;2/y7;2/y8;2/y9;2/y10;2/y11;2/y12;2/y13;2/y14

**FSCLLLLTGEIDLPVK=CLLLLTGEI**	1:F006-021-2:F008-016	N.D.	22.5	4	683.8488	2731.3633	52020	19	1/b4-2/y9;1/b5-2/y9;1/y3;1/y4;1/y6;1/y9-2/b13;1/y9-2/b12;1/y9-2/y15;1/y9-2/b14;1/y9-2/b6;1/y16-2/b8;1/y16-2/b3;2/b2;2/y2;2/y3;2/y6;2/y9;2/y10;2/y12
**PRGFSCLLLLTGEIDLPVK=CLLLLTG**	1:F003-021-2:F008-014	N.D.	22.5	3	934.1256	2799.353	42594	20	1/y2;1/y3;1/y5;1/y6;1/y7-2/y14;1/y7-2/y17;1/y7-2/b11;1/y7-2/b9;1/y7-2/b8;1/y7-2/y18;2/b2;2/b3;2/b4;2/b5;2/y2;2/y3;2/y6;2/y9;2/y10;2/y12
**GFSCLL=APRGFSCLLLLTGEIDLPVK**	1:F002-021-2:F005-010	N.D.	22.5	3	926.7966	2777.366	38857	18	1/b2;1/b3;1/b4;1/b6;1/y2;1/y3;1/y6-2/b15;1/y6-2/b11;1/y6-2/b12;1/y6;1/y10;1/y12;1/y16-2/y6;1/y17-2/y6;1/y20-2/y3;2/b2;2/b3;2/y2
**MAPRGFSCLLLLTGEIDLPVKRRA**	1:F001-024*	Oxidation C(1), Oxidation M(1)	23.5	4	672.8715	2687.4543	38795	11	b8*;b9*;b10*;b11*;y7;y9;y11;y12;y13;y14;y16
**EIDLPVKRRA**	1:F015-024	N.D.	22.5	2	598.859	1195.7021	38403	12	b1;b2;b3;b4;b5;b6;b7;b9;y2;y3;y4;y5
**APRGFSCLLLLTGEIDLPVKRR**	1:F002-023	N.D.	22.5	4	614.355	2453.3882	36440	23	b2;b3;b4;b6;b7;b11;b12;b14;b15;b16;y1;y2;y3;y4;y5;y7;y8;y9;y10;y12;y13;y14;y17
**GFSCLLLLTGEIDL=APRGFSCLL**	1:F002-010-2:F005-018	N.D.	22.5	4	614.355	2453.3882	36440	18	1/b2;1/b3;1/b4;1/b6;1/y2;1/y9-2/b12;1/y9-2/b8;1/y9-2/y13;1/y9-2/b9;1/y14-2/y4;2/b2;2/b3;2/y2;2/y3;2/y4;2/y6;2/y7;2/y8
**APRGFSCLL=CLLLLTGEIDLPVKRRA**	1:F002-010-2:F008-024	N.D.	22.5	3	957.4662	2869.375	35924	21	1/b2;1/b3;1/b3-2/y9;1/b4;1/b4-2/y9;1/b6;1/y2;1/y9-2/b2;1/y9-2/b7;1/y9-2/b1;1/y9-2/b14;1/y9-2/b5;1/y9-2/b6;1/y9-2/b16;2/y2;2/y3;2/y4;2/y5;2/y12;2/y14;2/y15
**APRGFSCLLLLTGEIDLPVKR=CLLL**	1:F002-022-2:F008-011	N.D.	22.5	3	919.4655	2755.3728	33480	21	1/b2;1/b3;1/b4;1/b6;1/b9-2/y4;1/y1;1/y2;1/y3;1/y4-2/b14;1/y4-2/b7;1/y4-2/b8;1/y4-2/b19;1/y4-2/y16;1/y4-2/b11;1/y4-2/y17;1/y4-2/b12;1/y4-2/b10;1/y9;1/y11;1/y14;2/y2

**IDLPVKRRA**	1:F016-024	N.D.	22.5	2	534.3372	1066.6584	32544	10	b2;b3;b4;b5;b6;b8;y2;y3;y4;y5
**RGFSC=FSCLLLLTGEIDLPVKRRA**	1:F004-008-2:F006-024	N.D.	22.5	5	542.8846	2709.3833	28739	17	1/b2;1/y2;1/y3;1/y4;1/y5-2/b10;1/y5-2/b4;1/y5;1/y5-2/b8;1/y5-2/b9;1/y12;1/y14;1/y15;1/y18-2/y5;2/b1;2/b2;2/b3;2/b4
**DLPVKRRA**	1:F017-024	N.D.	22.5	2	477.7956	953.5754	27883	7	b2;b3;b4;y2;y3;y4;y5
**GFSCLLL=FSCLLLLTGEIDLPVKRR**	1:F005-011-2:F006-023	N.D.	22.5	3	941.4539	2821.3379	24877	24	1/b2;1/b16-2/y7;1/y1;1/y2;1/y3;1/y4;1/y5;1/y7-2/b6;1/y7-2/b7;1/y7;1/y7-2/b3;1/y7-2/b5;1/y7-2/b8;1/y7-2/b4;1/y8;1/y9;1/y10;1/y12;1/y13;1/y14;1/y18-2/b4;2/b2;2/b3;2/y2
**GFSCLLLLTGEID=RGFSCLLLLTGEID**	1:F004-017-2:F005-017	N.D.	22.5	3	972.1198	2913.3354	24619	12	1/b2;1/b3;1/y2;1/y3;1/y7;2/b2;2/b3;2/b4;2/y6;2/y10-1/y14;2/y13-1/b5;2/y13-1/b13
**MAPRGFSCLLLLTG=CLLLLTGEIDL**	1:F001-014-2:F008-018	N.D.	21.9	3	893.4889	2677.4429	24322	6	1/y11-2/y13;2/b2;2/b3;2/b4;2/b5;2/b7
**APRGFSCLLLLTGEIDLPVKRRA**	1:F002-024	N.D.	22.5	4	632.1148	2524.4275	24126	18	b2;b3;b4;b6;b11;b12;b14;b15;b16;y2;y3;y4;y5;y12;y14;y15;y17;y18
**MAPRGFSCLLLL=FSCLLLLTGEIDLP**	1:F001-012-2:F006-019	N.D.	22.5	3	951.1242	2850.3489	23441	21	1/b2;1/b10-2/y14;1/y2;1/y3;1/y5-2/y14;1/y6;1/y7-2/y14;1/y7;1/y8;1/y9;1/y12-2/b11;1/y12-2/b12;1/y12-2/b6;2/b2;2/b3;2/b4;2/b5;2/b6;2/b7;2/y2;2/y4
**RGFSCLLLL=MAPRGFSCLLLLTGEI**	1:F001-016-2:F004-012	N.D.	22.5	3	913.8018	2738.3816	23023	20	1/b2;1/b3;1/b4;1/b5;1/b6;1/b7;1/y3;1/y4;1/y6;1/y9-2/b11;1/y9-2/b13;1/y9-2/b8;1/y9-2/y9;1/y16-2/y6;2/b1;2/b2;2/b3;2/b4;2/y2;2/y4
**GFSCLLLL=FSCLLLLTGEIDLPVKRR**	1:F005-012-2:F006-023	N.D.	22.5	3	979.1185	2934.3318	19996	26	1/b2;1/y1;1/y2;1/y3;1/y4;1/y5;1/y7;1/y8-2/b9;1/y8-2/b5;1/y8;1/y8-2/b4;1/y8-2/b3;1/y8-2/b8;1/y8-2/b15;1/y8-2/b6;1/y9;1/y10;1/y11;1/y12;1/y13;1/y14;1/y18-2/b4;2/b2;2/b3;2/y2;2/y4
**GFSCLLLLTGEIDLPVKRR=APRGFSC**	1:F002-008-2:F005-023	N.D.	22.5	3	955.4537	2863.3372	19884	24	1/b2;1/b3;1/b4;1/b6;1/y7-2/b11;1/y7-2/b5;1/y7-2/b10;1/y7-2/b4;1/y7-2/b9;1/y7-2/y16;2/b2;2/b3;2/y1;2/y2;2/y3;2/y4;2/y5;2/y7;2/y8;2/y9;2/y10;2/y12;2/y13;2/y

									14
**LLLTGEIDLPVKRRA**	1:F010-024	N.D.	22.5	2	847.5186	1693.0212	19437	16	b2;b3;b4;b5;b6;b9;b10;b11;b13;b14;y2;y3;y4;y5;y12;y14
**APRGFSCLLLL=MAPRGFSCLLLLTGE**	1:F001-015-2:F002-012	N.D.	22.5	3	932.1207	2793.3381	16728	19	1/b2;1/b3;1/b4;1/b6;1/y2;1/y4;1/y5-2/y15;1/y7-2/y15;1/y11-2/y11;1/y11-2/b13;2/b2;2/b3;2/b4;2/b5;2/b6;2/y2;2/y3;2/y4;2/y6
**MAPRGFSCLLLLTGEIDLPVKRR=CL**	1:F001-023-2:F008-009	N.D.	22.5	3	939.7855	2816.3325	16706	23	1/y2-2/b12;1/y2-2/b10;1/y2-2/b21;1/y2-2/y18;1/y2-2/b8;1/y2-2/b13;2/b2;2/b3;2/b4;2/b5;2/b6;2/y1;2/y2;2/y3;2/y4;2/y5;2/y7;2/y8;2/y9;2/y10;2/y12;2/y13;2/y14
**APRGFSCLLLLTG**	1:F002-014	N.D.	28.1	1	1347.8843	1346.8763	16614	5	b4;b7;y4;y6;y7
**MAPRGFSCLLLL**	1:F001-012*	Oxidation M(1)	22.5	2	668.8843	1335.7527	16532	7	b3*;b4*;b10*;y2;y4;y5;y10
**APRGFSCLLLLTGEIDLPVKRRA**	1:F002-024	N.D.	21.9	4	632.1149	2524.4277	16143	18	b4;b6;b7;b8;b9;b10;b11;b22;y2;y6;y7;y8;y9;y10;y13;y14;y15;y22
**PRGFSCLLLLTG=PRGFSCLLLLTGEID**	1:F003-014-2:F003-017	N.D.	22.5	3	969.779	2906.3132	15813	17	1/b3;1/b4;1/b5;1/b10-2/y12;1/y2;1/y3;1/y5;1/y6;1/y12-2/b6;1/y12-2/b11;1/y15-2/b8;1/y15-2/b6;2/y2;2/y3;2/y6;2/y7;2/y8
**APRGFSCLLLLTGEIDLPVKR=CLLLLT**	1:F002-022-2:F008-013	N.D.	22.5	3	990.7751	2969.3015	15495	19	1/b3;1/b4;1/b6;1/y1;1/y2;1/y3;1/y6-2/y19;1/y6-2/y16;1/y6-2/b11;1/y6-2/b10;1/y6-2/b9;1/y9;1/y11;1/y14;2/y1;2/y2;2/y3;2/y4;2/y5
**RGFSCLLLLTGEID=RGFSCLLLLTGE**	1:F004-015-2:F004-017	N.D.	22.5	3	948.1182	2841.3308	15283	17	1/b1;1/y2;1/y3;1/y4;1/y6;2/b2;2/b3;2/b4;2/y2;2/y3;2/y6;2/y7;2/y8;2/y12-1/b10;2/y12-1/b6;2/y12-1/b13;2/y14-1/b8
**MAPRGFSCLLLLTGEID=CLLLLTGE**	1:F001-017-2:F008-015	N.D.	21.9	4	674.3597	2693.407	15033	8	1/b7-2/y17;1/y8-2/y16;1/y8-2/b11;2/b2;2/b3;2/b4;2/b5;2/b7
**RGFSCLLLLTGEIDLPVKRR=FSCL**	1:F004-023-2:F006-009	N.D.	22.5	3	918.1212	2751.3396	14935	22	1/b2;1/y4-2/b18;1/y4-2/b5;1/y4-2/b17;1/y4-2/b10;1/y16-2/y4;2/b1;2/b2;2/b3;2/b4;2/y1;2/y2;2/y3;2/y4;2/y5;2/y7;2/y8;2/y9;2/y10;2/y12;2/y13;2/y14

**GFSCLLLLTGEIDLPVKRR=APRGFSCL**	1:F002-009-2:F005-023	N.D.	22.5	3	993.1191	2976.3335	14257	21	1/b3;1/b4;1/b6;1/y8-2/b4;1/y8-2/b16;1/y8-2/y18;1/y8-2/b9;1/y8-2/b10;2/b2;2/b3;2/y1;2/y2;2/y3;2/y4;2/y5;2/y7;2/y8;2/y9;2/y12;2/y13;2/y14
**GFSCLLLLTGEIDLPVKRR=GFSCLLL**	1:F005-011-2:F005-023	N.D.	22.5	3	960.4471	2878.3174	12406	21	1/b2;1/y2;2/b3;2/y1;2/y2;2/y3;2/y4;2/y5;2/y7-1/b7;2/y7-1/b8;2/y7;2/y7-1/b4;2/y7-1/b5;2/y7-1/b12;2/y7-1/b10;2/y8;2/y9;2/y10;2/y12;2/y13;2/y14
**CLLLLTGEIDL**	1:F008-018*	Oxidation C(1)	22.5	2	609.8526	1217.6893	12399	13	b3*;b4*;b5*;b6*;b7*;b8*;b10*;y2;y3;y4;y6;y7;y8
**TGEIDLPVKRRA**	1:F013-024	N.D.	21.9	2	677.8922	1353.7686	12169	12	b2;b3;b4;b5;b6;b11;y2;y6;y7;y8;y9;y10
**MAPRGFSCLLLLTGEIDLPVKRRA**	1:F001-024	N.D.	23.6	5	532.1014	2655.4675	12162	9	b4;b9;y10;y11;y12;y13;y14;y15;y22
**RGFSCLLLL**	1:F004-012	N.D.	22.5	1	1021.4427	1020.4348	11792	8	b2;b3;b4;b5;b8;y2;y4;y5
**GFSCLLLLTGE=GFSC**	1:F005-008-2:F005-015	N.D.	22.5	2	781.9707	1561.9255	11010	9	1/b3;1/b7-2/y4;1/y4-2/b10;1/y4-2/b6;1/y4-2/b8;2/y2;2/y3;2/y4;2/y6
**PRGFSCLLLLTGEIDLPVKR=SCLLLL**	1:F003-022-2:F007-012	N.D.	22.5	3	962.4517	2884.3313	10944	17	1/b3;1/b4;1/b5;1/y1;1/y2;1/y3;1/y6-2/b12;1/y6-2/b11;1/y6-2/b6;1/y6-2/b9;1/y6-2/b8;1/y6-2/b14;1/y9;1/y11;1/y14;2/y2;2/y4
**APRGFSCLLLL=MAPRGFSCLLLLTG**	1:F001-014-2:F002-012	N.D.	22.5	5	533.9536	2664.728	10928	16	1/b2;1/b3;1/b4;1/b6;1/y2;1/y4;1/y11-2/y12;1/y11-2/b13;2/b2;2/b3;2/b5;2/b6;2/y2;2/y3;2/y5;2/y6
**RGFSC=FSCLLLLTGEIDLPVKRRA**	1:F004-008-2:F006-024	N.D.	22.5	3	904.1339	2709.3777	10727	16	1/b2;1/y3;1/y4;1/y5-2/b10;1/y5-2/b4;1/y5;1/y5-2/b8;1/y5-2/b9;1/y5-2/b17;1/y12;1/y14;1/y15;2/b1;2/b2;2/b3;2/b4
**APRGFSCLLLL=FSCLLLLTGEIDLPVK**	1:F002-012-2:F006-021	N.D.	22.5	3	983.1032	2946.2859	10600	14	1/b2;1/b3;1/b4;1/b6;1/y2;1/y4;1/y8-2/y16;1/y11-2/b5;1/y16-2/b8;1/y16-2/b7;2/b2;2/y3;2/y10;2/y12
**SCLLLLTGEI=SCLLLLTGEIDLPVKRRA**	1:F007-016-2:F007-024	N.D.	22.5	3	1019.0975	3054.2688	10586	14	1/y3;1/y4;1/y6;1/y10-2/b8;1/y10-2/b9;1/y10-2/b4;1/y10-2/b6;1/y10-2/b7;2/y3;2/y4;2/y5;2/y12;2/y14;2/y15
**MAPRGFSCLL= MAPRGFSCLLLLTGEIDL**	1:F001-010-2:F001-018	N.D.	22.5	3	1014.1144	3039.3196	9817	15	1/b2;1/y2;1/y3-2/y18;1/y9-2/y18;1/y10-2/b8;1/y10-2/y16;2/b3;2/b5;2/b6;2/y2;2/y3;2/y4;2/y

									6;2/y7;2/y8
**GFSCLLLLTGEIDLPVKRR=GFSCLLLL**	1:F005-012-2:F005-023	N.D.	22.5	3	998.1039	2991.2881	9790	23	1/b2;1/y2;1/y4;1/y8-2/b15;1/y8-2/b6;1/y8-2/b7;1/y8-2/b8;1/y8-2/b10;1/y8-2/b4;1/y8-2/b11;1/y19-2/b7;2/b3;2/y1;2/y2;2/y3;2/y4;2/y5;2/y7;2/y9;2/y10;2/y12;2/y13;2/y14
**APRGFSCLLLLTGEI**	1:F002-016	N.D.	22.5	2	795.4114	1588.807	9764	12	b2;b3;b4;b6;b11;b12;b14;y3;y4;y6;y12;y14
**APRGFSCLLLLTGEIDLPVKRR=FSCLLL**	1:F002-023-2:F006-011	N.D.	22.5	3	1049.4358	3145.2834	9612	26	1/b2;1/b3;1/b4;1/b6;1/y1;1/y2;1/y3;1/y4;1/y5;1/y6-2/b14;1/y6-2/b10;1/y6-2/b7;1/y6-2/b21;1/y6-2/b16;1/y6-2/b12;1/y7;1/y9;1/y10;1/y11;1/y12;1/y13;1/y14;1/y22-2/b5;1/y22-2/b3;2/b2;2/y2
**APRGFSCLLLLTGEIDLPVKRR=FSCLL**	1:F002-023-2:F006-010	N.D.	22.5	3	1011.7664	3032.2754	9520	27	1/b2;1/b3;1/b4;1/b6;1/y1;1/y2;1/y3;1/y4;1/y5-2/y17;1/y5-2/b15;1/y5-2/b11;1/y5-2/b13;1/y5;1/y5-2/y19;1/y5-2/b21;1/y5-2/b8;1/y5-2/b17;1/y7;1/y9;1/y10;1/y12;1/y13;1/y14;1/y22-2/b3;1/y22-2/y4;2/b2;2/y2
**PVKRRA**	1:F019-024	N.D.	22.5	1	726.4699	725.462	9246	5	b3;b5;y3;y4;y5
**FSC=RGFSCLLLLTGEIDLPVKRRA**	1:F004-024-2:F006-008	N.D.	22.1	4	678.3609	2709.4119	9122	9	2/y2;2/y6;2/y7;2/y8;2/y9;2/y10;2/y13;2/y14;2/y15
**RGFSCLLLLT=PRGFSCLLLLTGEIDLP**	1:F003-019-2:F004-013	N.D.	22.5	3	988.4335	2962.2766	8979	19	1/b3;1/b14-2/y10;1/y3;1/y6;1/y7;1/y8;1/y9;1/y10-2/b8;1/y17-2/b6;1/y17-2/y7;2/b1;2/b2;2/b3;2/b4;2/y1;2/y2;2/y3;2/y4;2/y5
**RGFSCLLLLTGEIDLPVKRRA=SC**	1:F004-024-2:F007-008	N.D.	22.5	4	641.6015	2562.3743	8899	19	1/b1;1/b2;1/b3;1/b4;1/y2-2/b14;1/y2-2/b9;1/y2-2/b15;1/y2-2/b12;1/y2-2/b7;1/y2-2/b13;1/y2-2/b8;1/y2-2/b10;1/y3;1/y4;1/y5;1/y12;1/y14;1/y15;1/y20-2/y2
**EIDLPV**	1:F015-020	N.D.	22.5	1	685.3515	684.3436	8683	5	b1;b2;b3;b4;b5
**PRGFSCLLLLTGEIDLPVKRRA**	1:F003-024	N.D.	21.7	4	614.3555	2453.3904	8414	7	b7;b8;b9;y7;y11;y12;y14
**MAPRGFSCLLLLTGE=FSCLLLLTGE**	1:F001-015-2:F006-015	N.D.	21.9	3	900.8144	2699.4194	8350	5	2/b2;2/b3;2/b4;2/b5;2/b7

**SC=CLLLLTG**	1:F007-008-<br>2:F008-014	N.D.	22.5	1	938.4548	937.4469	8111	6	1/y1-2/y7;1/y2-2/b5;1/y2;1/y3;1/y5;1/y6
**RGFSCLLLLTGEID=RGFSCLLLLT**	1:F004-013-<br>2:F004-017	N.D.	22.5	5	532.0209	2655.0649	7932	18	1/b1;1/b4;1/y1;1/y2;1/y3;1/y4;1/y5;2/b2;2/b3;2/y2;2/y3;2/y6;2/y7;2/y8;2/y10-1/b12;2/y10-1/b6;2/y10-1/b5;2/y14-1/b8
**GEIDLPVKRRA**	1:F014-024	N.D.	21.9	2	627.3687	1252.7214	7832	10	b2;b3;b4;b5;b7;y2;y6;y8;y9;y10
**PRGFSCLLLLTGE=FSCLLLLTGEIDL**	1:F003-015-<br>2:F006-018	N.D.	22.5	3	947.1171	2838.3276	7724	18	1/b2;1/y2;1/y3;1/y4;1/y6;1/y7;1/y8;1/y13-2/b9;1/y13-2/b4;1/y13-2/b8;1/y13-2/b12;1/y13-2/b3;1/y13-2/b5;2/b3;2/y2;2/y3;2/y4;2/y6
**GFSCLLLLTGEID=<br>MAPRGFSCLLLLTGE**	1:F001-015-<br>2:F005-017	N.D.	22.5	3	995.7682	2984.281	7464	20	1/b3;1/y2;1/y3;1/y6;1/y7;1/y8;1/y13-2/b11;1/y13-2/b8;1/y13-2/b14;1/y13-2/b13;1/y14-2/y13;1/y15-2/b7;2/b2;2/b3;2/b5;2/b6;2/y2;2/y3;2/y4;2/y6
**FSCLLLLT=FSCLLLLTGEIDLPVKRRA**	1:F006-013-<br>2:F006-024	N.D.	22.5	3	1017.4348	3049.2805	6978	17	1/b2;1/y1;1/y2;1/y3;1/y4;1/y5;1/y8-2/b14;1/y8-2/b7;1/y8-2/b6;1/y8-2/b8;1/y8-2/b3;2/y3;2/y4;2/y5;2/y12;2/y14;2/y15
**RGFSCLLLLTGEIDLPVKRR=SC**	1:F004-023-<br>2:F007-008	N.D.	22.5	4	623.8429	2491.3398	6881	24	1/b1;1/b2;1/b3;1/b4;1/y1;1/y2-2/b14;1/y2-2/b9;1/y2-2/b15;1/y2-2/b12;1/y2-2/b7;1/y2-2/b13;1/y2;1/y2-2/b8;1/y2-2/b10;1/y3;1/y4;1/y5;1/y7;1/y9;1/y10;1/y11;1/y12;1/y14;1/y18-2/y2
**LPVKRRA**	1:F018-024	N.D.	22.5	2	420.2817	838.5475	6841	8	b2;b3;b4;b5;b6;y3;y4;y5
**PRGFSCLLLLTGEIDLPVKR=CLLLLTG**	1:F003-022-<br>2:F008-014	N.D.	22.5	3	986.1235	2955.3467	6680	17	1/y2;1/y3;1/y5;1/y6;1/y7-2/y19;1/y7-2/b11;1/y7-2/b9;1/y7-2/b8;1/y7-2/y18;1/y7-2/b10;2/b3;2/y1;2/y2;2/y3;2/y9;2/y11;2/y14
**MAPRGFSCL=MAPRGFSCLLLLTGEIDL**	1:F001-009-<br>2:F001-018	N.D.	22.5	3	976.4437	2926.3071	6478	11	1/b3;1/b5;2/b6;2/y2;2/y3;2/y4;2/y6;2/y7;2/y8;2/y9-1/b9;2/y9-1/b8
**FSCLLLLTGEIDLPVKRR=CLLLLTGE**	1:F006-023-<br>2:F008-015	N.D.	22.5	3	977.7795	2930.3147	6414	24	1/b5-2/y8;1/b6-2/y8;1/y2;1/y3;1/y4;1/y6;1/y8-2/b14;1/y8-2/b16;1/y8-2/b13;1/y8-2/b8;1/y8-2/b7;1/y8-2/b17;1/y18-2/b1;2/b2;2/y1;2/y2;2/y3;2/y4;2/y5;2/y7

									;2/y9;2/y10;2/y12;2/y14
**LTGEIDLPVKRRA**	1:F012-024	N.D.	21.9	2	734.4342	1466.8525	6376	8	b2;b4;b6;y2;y6;y8;y9;y10
**RGFSCLLLLTGE= SCLLLLTGEIDLPVKR**	1:F004-015-2:F007-022	N.D.	22.5	3	1025.756	3074.2441	6215	20	1/b1;1/b2;1/b3;1/b4;1/y2;1/y3;1/y4;1/y6;1/y9-2/y16;1/y12-2/b15;1/y12-2/b2;1/y12-2/b6;1/y12-2/b3;1/y12-2/b5;2/y1;2/y2;2/y3;2/y9;2/y11;2/y14
**TGEIDLPVKRRA**	1:F013-024	N.D.	22.1	2	677.8928	1353.7698	5915	8	b2;b3;b4;y2;y6;y8;y9;y10
**MAPRGFSCLLLLTGEIDLPVKR=CLL**	1:F001-022-2:F008-010	N.D.	22.5	3	925.455	2773.3411	5814	12	1/y2;1/y3-2/b11;1/y3-2/b9;1/y3-2/y19;2/b3;2/b5;2/b6;2/y2;2/y3;2/y9;2/y11;2/y14
**APRGFSCLLLLTGEIDLPVK=CLLLL**	1:F002-021-2:F008-012	N.D.	22.5	3	905.136	2712.3843	5753	15	1/b2;1/b4;1/y3;1/y5-2/y18;1/y5-2/b12;1/y5-2/b19;1/y5-2/b10;1/y5-2/b11;1/y5-2/b13;1/y5-2/b9;1/y10;1/y12;1/y20-2/b3;2/y2;2/y4
**GFSCLLLLTGEID=PRGFSCLLLLTGEID**	1:F003-017-2:F005-017	N.D.	22.5	3	1004.4337	3010.2773	5753	8	1/b2;1/b3;1/y2;2/b3;2/y3;2/y6;2/y7;2/y8
**SCL=CLLLLT**	1:F007-009-2:F008-013	N.D.	22.5	1	994.4475	993.4396	5635	6	1/y1;1/y2;1/y3-2/b4;1/y3;1/y4;1/y5
**FSC=CLLLLTGEID**	1:F006-008-2:F008-017	N.D.	22.5	2	721.8849	1441.7539	5494	8	1/y2;1/y3-2/b6;1/y3;1/y6;1/y7;1/y8;1/y10-2/y2;2/b2
**PRGFSCLLLLT**	1:F003-013*	Oxidation C(1)	22.5	2	618.3611	1234.7064	5130	13	b3;b8*;b9*;b10*;y1;y2;y3;y4;y5;y6*;y7*;y8*;y9*
**RGFSCLLL=SCLLLLTGEIDLPVKRRA**	1:F004-011-2:F007-024	N.D.	22.5	3	968.1096	2901.3049	5055	17	1/b1;1/b2;1/b3-2/y8;1/b3;1/b4;1/y2;1/y8-2/b5;1/y8-2/b4;1/y8-2/b10;1/y8-2/b9;1/y8-2/b2;1/y8-2/b8;1/y18-2/b5;2/y4;2/y12;2/y14;2/y15

*The symbol “=” represents the dimerization via disulfide bonds between two peptide sequences. The first row is the dimerized HNG peptide while the remaining sequences are the dimerized HNG fragments. RT; Retention time, Min; Minutes.
